# Perioperative Risk Stratification in Surgically Treated Endometrial Cancer: The Impact of Nutritional Status and Comorbidity Burden on Morbidity and Survival

**DOI:** 10.1245/s10434-026-19906-5

**Published:** 2026-06-02

**Authors:** Murat Cengiz, Onur Can Zaim, Bilal Esat Temiz, Anil Karakilinc, Omer Alp Yavuz, Hasan Volkan Ege, Utku Akgor, Murat Gultekin, Derman Basaran

**Affiliations:** 1 Department of Obstetrics and Gynecology, Division of Gynecologic Oncology, Faculty of Medicine, Hacettepe University, Ankara, Turkey; 2 Department of Obstetrics and Gynecology, Faculty of Medicine, Hacettepe University, Ankara, Turkey; 3 Department of Gynecologic Oncology, Etlik City State Hospital, Ankara, Turkey

**Keywords:** Endometrial Neoplasms, Aged, Comorbidity, Nutritional status, Survival rate, Morbidity

## Abstract

**Background:**

To compare outcomes between older (≥ 70 years) and younger (< 70 years) patients with surgically treated endometrial cancer, evaluating the Prognostic Nutritional Index (PNI), Systemic Immune-Inflammation Index (SII), and Age-Adjusted Charlson Comorbidity Index (ACCI).

**Methods:**

This retrospective cohort study included 319 patients who underwent surgery at a tertiary center (2014–2024). Demographics, pathology, treatment, and outcomes were analyzed. Perioperative morbidity was graded (Clavien–Dindo). Frailty (ACCI), PNI, and SII were assessed. Overall survival and disease-specific survival were analyzed in parallel; Kaplan–Meier analyses were performed overall and stratified by stage and histology. Major morbidity was analyzed with multivariable logistic regression.

**Results:**

Of 319 patients, 93 (29.2%) were aged ≥ 70 years. Older patients more frequently exhibited high-grade histology, advanced stage, ASA III–IV status, high ACCI, and low PNI. Admissions to the intensive care unit were higher in older patients, but major morbidity (4.3% vs. 3.5%; *p* = 0.60) and 30-day mortality (1.1% vs. 0.9%; *p* = 0.60) were comparable between groups. Low PNI demonstrated moderate discriminatory ability for major complications. In multivariable analysis, low PNI (odds ratio 5.78; *p* = 0.012) and ASA III–IV (odds ratio 3.89; *p* = 0.047) were independently associated with major morbidity, whereas age ≥ 70 was not. For overall survival, higher PNI remained protective (hazard ratio 0.48 per 5-point increase; *p* < 0.001) and ASA III–IV predicted worse outcomes (hazard ratio 3.80; *p* = 0.002); however, chronological age was not an independent predictor.

**Conclusion:**

Older age is associated with more aggressive tumor biology but not with higher rates of major complications. PNI and ASA class are independent predictors of major morbidity and survival and should be integrated into perioperative risk stratification rather than using chronological age alone.

**Supplementary Information:**

The online version contains supplementary material available at 10.1245/s10434-026-19906-5.

Endometrial cancer is the sixth most common malignancy among women worldwide, with over 420,000 new cases and nearly 100,000 related deaths reported annually according to GLOBOCAN estimates.^1^ With increasing life expectancy, the incidence of endometrial cancer in older populations has been rising.^2^ This demographic shift necessitates a deeper understanding of the specific challenges associated with the management of endometrial cancer in older patients.

Surgical intervention remains the cornerstone of treatment for endometrial cancer.^3^ However, older patients frequently present with multiple comorbidities, frailty, and age-related physiological changes that can significantly affect both perioperative and postoperative management. These factors may increase the risk of morbidity and influence overall treatment outcomes. Despite these concerns, limited data exist regarding the prognostic indicators and perioperative risk factors specific to this population.^4^

Nutritional status is a key determinant of postoperative recovery and overall prognosis in patients with cancer. Numerous studies have demonstrated that malnutrition is closely associated with increased postoperative complications, delayed recovery, and poorer survival outcomes.^5,6^ In geriatric patients, nutritional deficiencies are particularly prevalent, exacerbating postoperative challenges. These complications are not solely attributable to aging but are significantly influenced by malnutrition and systemic inflammation, as reflected by indices such as the Prognostic Nutritional Index (PNI) and the Systemic Immune-Inflammation Index (SII).^7,8^ Both PNI and SII have emerged as reliable prognostic markers that offer valuable insights into perioperative risk and long-term survival.^9^ The Age-Adjusted Charlson Comorbidity Index (ACCI), which incorporates patient age as a correction factor in the Charlson Comorbidity Index, has been established as a valuable tool for predicting both short- and long-term outcomes in various cancers.^10^ Numerous studies have demonstrated that ACCI plays a significant role in multiple aspects of the care of older patients with cancer, including treatment selection, response to therapy, tumor progression, morbidity, and survival outcomes.^11^ Collectively, these indices highlight the need for comprehensive risk assessment to optimize care in this vulnerable population.

Comparative studies evaluating clinicopathologic characteristics, perioperative outcomes, and survival between older and younger patients are essential to address these challenges and guide individualized management.^12^ The age threshold of 70 years was chosen on the basis of both institutional and literature precedent. International Society of Geriatric Oncology generally define older adults in oncologic settings from approximately 65–70 years onward, and the National Comprehensive Cancer Network Older Adult Oncology framework uses 70 years as a pragmatic threshold for geriatric assessment. Multiple prior endometrial cancer cohorts comparing surgical outcomes between older and younger women have likewise used a 70-year cutoff, which allows direct comparison with the existing literature.^5,13–15^ Therefore this study, aimed to evaluate the clinical and prognostic differences between older (≥ 70 years) and younger (< 70 years) patients with endometrial cancer. Specifically, we aimed to investigate the impact of age on perioperative morbidity, postoperative morbidity according to Clavien–Dindo, inflammatory and nutritional indices such as the SII and PNI, and overall survival (OS) outcomes. By identifying age-related disparities in treatment patterns and survival, this study seeks to provide insights that may inform more individualized management strategies for older patients with endometrial cancer.

## Materials and Methods

**Fig. 1:**
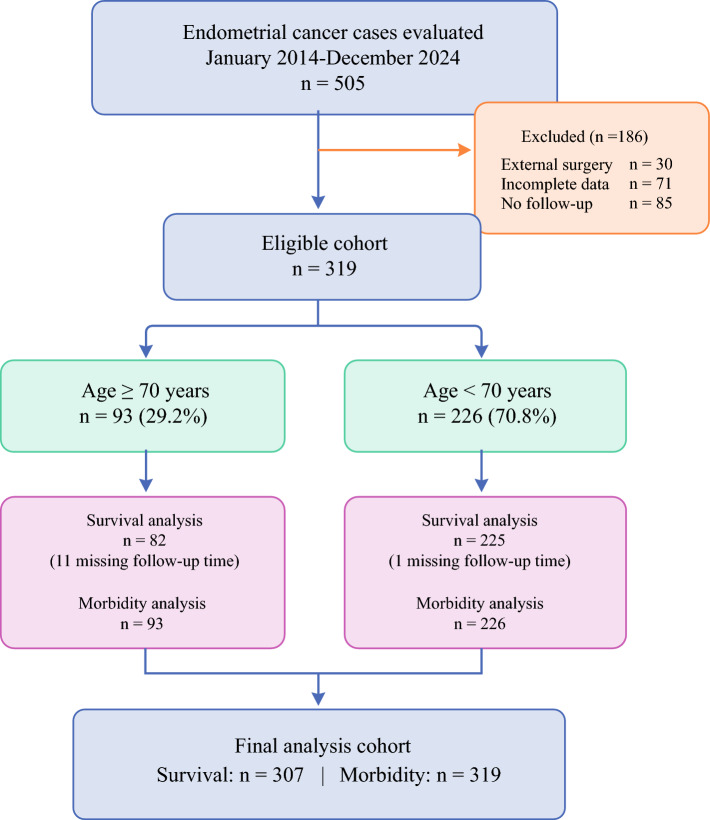
Study flowchart summarizing patient selection process

This retrospective cohort study was conducted at the Department of Obstetrics and Gynecology, Division of Gynecologic Oncology, Hacettepe University, Turkey, and included patients who underwent surgery for endometrial cancer between January 2014 and December 2024. Survival data were obtained for patients followed until September 2025. Eligible participants were those who received primary surgical treatment for histologically confirmed endometrial carcinoma at our institution during this period and had complete clinical and follow-up data available for analysis. Patients who underwent surgery at external centers, those with incomplete or missing data, and those who did not attend follow-up visits were excluded. The study population was stratified into two groups according to age: younger (< 70 years) and older (≥ 70 years). The study inclusion process is illustrated in Fig. 1. Demographic, clinical, and treatment-related data were collected from electronic medical records, including operative reports, pathology reports, comorbidities, and adjuvant treatments. Clinical and pathological staging followed the International Federation of Gynecology and Obstetrics (FIGO) 2009 staging system.^16^ Data on adjuvant chemotherapy and radiotherapy (modality, regimen, and completion) were uniformly abstracted for all patients; adjuvant therapy indications followed recommendations from the European Society of Medical Oncology, the European Society of Gyneacological Oncology, and the European Society of Radiotherapy and Oncology,^3^ and institutional multidisciplinary tumor board decisions, without age-based protocols.

Perioperative morbidity was classified according to the Clavien–Dindo classification. Comorbidity burden was assessed using the ACCI, calculated as originally described by Charlson et al.^17^. Based on the composite score incorporating age and baseline comorbidities, patients were stratified into three risk categories: low (0–1), moderate (2–3), and high (≥ 4).^18^ Preoperative physiological status was evaluated using the American Society of Anesthesiologists (ASA) physical status classification, and patients were dichotomized as ASA I–II or ASA III–IV for statistical analyses, with higher scores indicating greater systemic disease burden. Recurrence was defined as radiologically or histologically confirmed disease after completion of primary treatment.

The PNI was calculated as follows:^19,20^

$$\begin{aligned} &\left( {10 \times {\text{serum albumin}}\;\left[ {{\mathrm{g}}/{\mathrm{dL}}} \right]} \right) \\ &\quad+ \left( {0.005 \times {\text{total lymphocyte count}}\;\left[ {/{\mathrm{mm}}^{3} } \right]} \right) \end{aligned}$$

The SII was calculated as:^21^

$$\begin{aligned}&Platelet\,count \times neutrophil\,count/lymphocyte\,count,\\ &\quad\hbox{where all blood parameters were expressed as} \times 10^{9}/\hbox{L}. \end{aligned}$$

The PNI was analyzed both as a continuous variable and as a categorical variable. Continuous analyses were considered primary, to preserve the full prognostic information of the index. For clinical interpretability and risk stratification, PNI was additionally categorized using a threshold of 40, which has been among the most frequently reported cutoffs in oncologic and surgical literature.^20,22,23^ Accordingly, PNI values > 40 were classified as high and ≤ 40 as low. This cutoff was prespecified prior to outcome analyses.

All statistical analyses were performed using IBM SPSS Statistics for Windows, version 23 (IBM Corp., Armonk, NY, USA). Continuous variables were tested for normality using the Kolmogorov–Smirnov test. Normally distributed variables were compared using Student’s t-test, and non-normally distributed variables were compared using the Mann–Whitney U test. Categorical variables were compared using the chi-squared or Fisher’s exact test, as appropriate.

The association between PNI, SII, ACCI, ASA class and perioperative outcomes (major morbidity, and mortality) was evaluated using univariate and multivariate logistic regression analyses. A multivariable logistic regression model for major morbidity (Clavien–Dindo ≥ III) was constructed including age, disease stage, ASA class, and PNI; alternative models including ACCI and histologic grade were explored as sensitivity analyses. Given the limited number of major morbidity events (*n* = 14), model parsimony was prioritized to preserve an adequate events-per-variable ratio. Disease-specific survival (DSS) was defined as the time from surgery to cancer-related death. Cause of death was ascertained from hospital records and national death registry entries. Because a substantial proportion of deaths in this retrospective cohort had incomplete cause-of-death documentation, DSS events were pragmatically defined as (i) deaths explicitly attributed to endometrial cancer or (ii) deaths of unknown cause in patients with documented disease recurrence prior to death; non-cancer and unknown-without-recurrence deaths were censored at the date of death. DSS was analyzed as a secondary endpoint alongside OS. OS and DSS were calculated using the Kaplan–Meier method, and survival curves were compared with the log-rank test. To address the heterogeneous distribution of stage and histology across age groups, Kaplan–Meier analyses were also performed within prespecified strata (stage I–II vs. III–IV; low-grade vs. high-grade/non-endometrioid histology). Variables with *p* < 0.10 in univariate analysis were included in multivariate Cox proportional hazards models to estimate hazard ratios (HRs) and 95% confidence intervals (CIs). A two-tailed *p*-value < 0.05 was considered statistically significant.

The study protocol was approved by the Hacettepe University Ethics Committee (approval no: 2025/18-02). As this was a retrospective study, the requirement for informed consent was waived. The study was conducted in accordance with the Declaration of Helsinki.

## Results

During the study period, 505 consecutive patients with histologically confirmed endometrial carcinoma were evaluated at our institution. After excluding 30 patients who underwent primary surgery at external centers, 71 with incomplete clinical or pathological data, and 85 who did not attend any postoperative follow-up visit, a final cohort of 319 patients who underwent primary surgical treatment at our center was analyzed. Of these, 226 (70.8%) were aged < 70 years and 93 (29.2%) were aged ≥ 70 years a (Fig. 1). The clinicopathologic characteristics of the study population are summarized in Table 1.

**Table 1:** Baseline clinicopathologic characteristics by age group (<70 vs ≥70 years)

Variable	Age < 70 years	Age ≥ 70 years	Total	*p*-Value
*Histologic type*				**< 0.001**
Endometrioid grade 1–2	204 (90.3)	63 (67.7)	267 (83.7)	
Endometrioid grade 3	11 (4.9)	9 (9.7)	20 (6.3)	
Serous/clear cell	8 (3.5)	16 (17.2)	24 (7.5)	
Other	3 (1.3)	5 (5.4)	8 (2.5)	
*ACCI*				**< 0.001**
2–3 (intermediate risk)	178 (78.8)	46 (49.5)	224 (70.2)	
≥4 (high risk)	48 (21.2)	47 (50.5)	95 (29.8)	
*Stage*				**0.003**
I	158 (69.9)	49 (52.7)	207 (64.9)	
II	36 (15.9)	12 (12.9)	48 (15.0)	
III	26 (11.5)	23 (24.7)	49 (15.4)	
IV	6 (2.7)	9 (9.7)	15 (4.7)	
*ASA score*				**< 0.001**
1	94 (41.6)	5 (5.4)	99 (31.0)	
2	119 (52.7)	51 (54.8)	170 (53.3)	
3	11 (4.9)	36 (38.7)	47 (14.7)	
4	2 (0.9)	1 (1.1)	3 (0.9)	
*Prognostic nutritional index category*				**< 0.001**
Low ≤40	62 (27.4)	50 (53.8)	112 (35.1)	
High >40	164 (72.6)	43 (46.2)	207 (64.9)	
*Systemic inflammatory index category*				0.050
Low < 575	120 (53.1)	38 (40.9)	158 (49.5)	
High ≥575	106 (46.9)	55 (59.1)	161 (50.5)	
*Surgical procedure*				**< 0.001**
Minimal Invasive	89 (39.4)	18 (19.4)		
Laparatomy	137 (60.6)	75 (80.6)		
TAH + BSO + pelvic LND + omentectomy	25 (11.1)	12 (12.9)	37 (11.6)	
TAH + BSO + pelvic–paraaortic LND + omentectomy	56 (24.8)	29 (31.2)	85 (26.6)	
TLH + BSO + sentinel LND	54 (23.9)	8 (8.6)	62 (19.4)	
RA-TLH + BSO + sentinel LND	33 (14.6)	9 (9.7)	42 (13.2)	
VH + BSO	2 (0.9)	1 (1.1)	3 (0.9)	
Debulking	6 (2.7)	10 (10.8)	16 (5.0)	
TAH + BSO	50 (22.1)	24 (25.8)	74 (23.2)	

Data are presented as n (%). P-values are from chi-squared or Fisher’s exact tests where appropriate

Bold values indicate statistical significance (*p* < 0.05)

ACCI, Age-Adjusted Charlson Comorbidity; ASA, American Society of Anesthesiologists; BSO, bilateral salpingo-oophorectomy; LND, lymph node dissection; RA-TLH, Robotic assisted total laparoscopic hysterectomy; TAH, total abdominal hysterectomy; TLH, total laparoscopic hysterectomy; VH, vaginal hysterectomy

The distribution of ACCI risk differed significantly by age group: 50.5% of patients aged ≥ 70 years were in the high-risk category (ACCI ≥ 4), compared with 21.2% in those aged < 70 years (*p* < 0.001). Older patients also had a higher proportion of high-grade/non-endometrioid histology (32.3% vs. 9.7%, *p* < 0.001) and advanced-stage disease (stage III–IV: 34.4% vs. 14.2%, *p* = 0.003). ASA III–IV was observed in 39.8% of older versus 5.8% of younger patients (*p* < 0.001). Low PNI (≤ 40) was more prevalent in older patients (53.8% vs. 27.4%, *p* < 0.001), and high SII was more common in this group (59.1% vs. 46.9%, *p* = 0.050).

In early-stage (stage I–II) disease, the proportion of minimally invasive procedures was 39.7% (77/194) in patients aged < 70 years and 27.9% (17/61) in those aged ≥ 70 years (*p* = 0.13). In stage III–IV disease, minimally invasive surgery was used in 37.5% (12/32) of younger patients and 3.1% (1/32) of older patients (*p* = 0.001). The overall operative time had a median of 150 min (interquartile range [IQR] 100–180). Operations were longer in patients aged ≥ 70 years than in those aged < 70 years (mean rank 191.3 vs. 147.1; *p* < 0.001).

Postoperative outcomes are summarized in Table 2. Older patients had higher rates of admission to the intensive care unit (29.0% vs. 12.8%, *p* < 0.001) and of any-grade morbidity (84.9% vs. 64.2%, *p* < 0.001), and morbidity occurring within 30 days was also more frequent in this group (18.3% vs. 6.2%, *p* < 0.001). The distribution of Clavien–Dindo grades is detailed in Table 2. Major morbidity (Clavien–Dindo ≥III) occurred in 4.3% of older and 3.5% of younger patients (*p* = 0.60), and 30-day mortality was low and comparable (1.1% vs. 0.9%, *p* = 0.60). Among patients with any complication, the most frequent types were postoperative infection (*n* = 18; 9.7% in those aged ≥ 70 years vs. 4.0% in those aged < 70 years), acute kidney injury (*n* = 6; 4.3% vs. 0.9%), postoperative bleeding (*n* = 2), pulmonary embolism (*n* = 2), cerebrovascular accident (n=2), anastomotic leak (*n* = 2), and cardiac arrest (*n* = 1). The age-related difference in overall Clavien–Dindo grade distribution did not reach statistical significance (*χ*^2^ = 12.12, *df* = 6, *p* = 0.059).

**Table 2:** Postoperative outcomes, surgical morbidity, and adjuvant treatment by age group

Variable	< 70 years (*n* = 226)	≥ 70 years (*n* = 93)	Total (*N* = 319)	*p*-Value
*ICU requirement*				**< 0.001**
No	197 (87.2)	66 (71.0)	263 (82.4)	
Yes	29 (12.8)	27 (29.0)	56 (17.6)	
*Overall morbidity*				**< 0.001**
None	81 (35.8)	14 (15.1)	95 (29.8)	
Minor	137 (60.6)	75 (80.6)	212 (66.5)	
Major	8 (3.5)	4 (4.3)	12 (3.8)	
*Clavien–Dindo classification*				
Grade I	161 (71.2)	69 (74.2)	230 (72.1)	
Grade II	57 (25.2)	19 (20.4)	76 (22.8)	
Grade IIIa	0 (0.0)	1 (1.1)	1 (0.3)	
Grade IIIb	1 (0.4)	1 (1.1)	2 (0.6)	
Grade IVa	5 (2.2)	1 (1.1)	6 (1.8)	
Grade IVb	0 (0.0)	3 (3.2)	3 (0.9)	
Grade V	2 (0.9)	0 (0.0)	2 (0.6)	
*Complication type*				
No complication	210 (92.9)	76 (81.7)	286 (89.7)	
Cerebrovascular accident	1 (0.4)	1 (1.1)	2 (0.6)	
Pulmonary embolism	1 (0.4)	1 (1.1)	2 (0.6)	
Acute kidney injury	2 (0.9)	4 (4.3)	6 (1.9)	
Postoperative infection	9 (4.0)	9 (9.7)	18 (5.6)	
Anastomotic leak	1 (0.4)	1 (1.1)	2 (0.6)	
Postoperative bleeding	2 (0.9)	0 (0.0)	2 (0.6)	
Cardiac arrest	0 (0.0)	1 (1.1)	1 (0.3)	
*30-day mortality*				*0.600 (NS)*
No	224 (99.1)	92 (98.9)	316 (99.1)	
Yes	2 (0.9)	1 (1.1)	3 (0.9)	
*30-day morbidity*				***< 0.001***
No	212 (93.8)	76 (81.7)	288 (90.3)	
Yes	14 (6.2)	17 (18.3)	31 (9.7)	
*Recurrence*				***< 0.001***
No	207 (91.6)	59 (72.0)	266 (86.4)	
Yes	19 (8.4)	23 (28.0)	42 (13.6)	
*Postoperative chemotherapy*				***0.011***
No	181 (80.1)	62 (66.7)	243 (76.2)	
Yes	45 (19.9)	31 (33.3)	76 (23.8)	
*Postoperative radiotherapy*				*0.599 (NS)*
No	136 (60.2)	53 (57.0)	189 (59.2)	
Yes	90 (39.8)	40 (43.0)	130 (40.8)	

Data are presented as n (%). *p*-Values are from chi-squared or Fisher’s exact tests. Morbidity is classified according to the Clavien–Dindo grading system

Bold values indicate statistical significance (*p* < 0.05)

ICU, Intensive care unit; NS, not significant

**Fig. 2:**
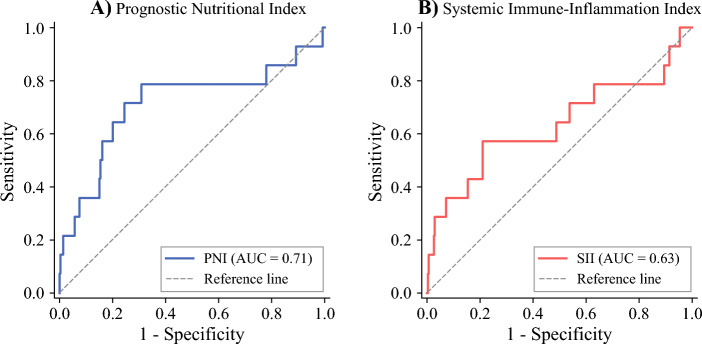
ROC curves for predicting major postoperative morbidity (Clavien–Dindo grade ≥III): (A) Prognostic Nutritional Index (PNI) and (B) Systemic Immune-Inflammation Index (SII). Because lower PNI values were associated with an increased risk of major morbidity, ROC analysis was constructed by defining lower PNI values as the positive test state. PNI demonstrated a moderate discriminatory ability for major morbidity (AUC = 0.71, 95% CI: 0.54–0.89, p = 0.008), with lower PNI values indicating increased risk. In contrast, SII showed limited discriminatory performance (AUC = 0.623, 95% CI 0.522–0.725, p = 0.012)

Major morbidity occurred in 8 of 95 (8.4%) patients with ACCI ≥ 4 and 6 of 224 (2.7%) with ACCI 2–3 (linear-by-linear trend *p* = 0.022). When stratified by age group, the rate of major morbidity in patients with ACCI ≥ 4 was 8.3% in those aged < 70 years (vs. 2.8% for ACCI 2–3; odds ratio [OR] 3.15, Fisher *p* = 0.099) and 8.5% in those aged ≥ 70 years (vs. 2.2%; OR 4.19, *p* = 0.36); the age × ACCI interaction term in a logistic model was non-significant (*p* = 0.83), indicating a homogeneous effect of comorbidity burden across age strata. The mean SII value was higher in patients with major morbidity than in those with minor morbidity (1393.27 vs. 728.56). However, the Mann–Whitney U test did not reveal a statistically significant difference between the groups (*U* = 1565.0, *p* = 0.091). Patients with a PNI ≤ 40 experienced a significantly higher rate of major morbidity than did those with PNI > 40 (9.8% vs. 1.4%, (*χ*^2^(1) = 12.14, *p* < 0.001). In receiver operating characteristic (ROC) analysis, using lower PNI values as the risk direction for major postoperative morbidity, the area under the curve was 0.71 (95% CI 0.54–0.89; *p* = 0.008) (Fig. 2). In multivariable logistic regression including age, stage, ASA class, and PNI, low PNI (OR 5.78; 95% CI 1.48–22.59; *p* = 0.012) and ASA III–IV (OR 3.89; 95% CI 1.02–14.90; *p* = 0.047) remained independent predictors of major morbidity, whereas age ≥ 70 years (OR 0.39, *p* = 0.19) and advanced stage (OR 2.37, *p* = 0.16) were not (Table 3).

**Table 3:** Univariate and multivariable logistic regression analysis for major surgical morbidity (Clavien–Dindo ≥III)

Variable	Univariate	*p*-Value	Multivariable	*p*-Value
	OR (95% CI)		OR (95% CI)	
Age ≥ 70 versus < 70	1.37 (0.45–4.20)	0.582	0.39 (0.09–1.59)	0.188
Stage III–IV versus I–II	3.19 (1.07–9.56)	**0.038**	2.37 (0.72–7.83)	0.157
High-grade versus low-grade	3.05 (0.98–9.50)	0.054	–	–
ASA III–IV versus I–II	4.57 (1.51–13.82)	**0.007**	3.89 (1.02–14.90)	**0.047**
ACCI ≥ 4 versus 2–3	3.34 (1.13–9.91)	**0.030**	–	–
PNI low (≤ 40) versus > 40	7.41 (2.02–27.14)	**0.003**	5.78 (1.48–22.59)	**0.012**
SII high versus low	1.81 (0.59–5.53)	0.297	–	–
MIS versus open surgery	0.53 (0.14–1.93)	0.334	–	–

The multivariable model was constructed a priori using clinically relevant variables and reviewer-requested covariates while maintaining model parsimony because of the limited number of major morbidity events. Total events = 14 (events per variable = 3.5). Bold values indicate statistical significance (p < 0.05)

ACCI, age-adjusted Charlson comorbidity index; ASA, American Society of Anesthesiologists; CI, confidence interval; MIS, minimally invasive surgery; OR, odds ratio; PNI, prognostic nutritional index; SII, systemic immune-inflammation index

Adjuvant chemotherapy and radiotherapy were given according to multidisciplinary tumor board decisions without age-based protocols. In early-stage disease, the use of chemotherapy (8.2% vs. 14.8%, *p* = 0.14) and radiotherapy (35.1% vs. 41.0%, *p* = 0.45) did not differ by age. In advanced-stage disease, chemotherapy was administered less frequently in older patients (68.8% vs. 90.6%, *p* = 0.060), whereas radiotherapy use did not differ (46.9% vs. 68.8%, *p* = 0.13). Recurrence occurred in 42 of 308 patients with available follow-up. Within stage I–II, recurrence rates were 6.7% (13/194) in patients aged < 70 years and 14.8% (8/54) in those aged ≥ 70 years (*p* = 0.09). Within stage III–IV, recurrence was substantially more frequent in older patients (53.6% [15/28] vs. 18.8% [6/32]; *p* = 0.007).

**Fig. 3:**
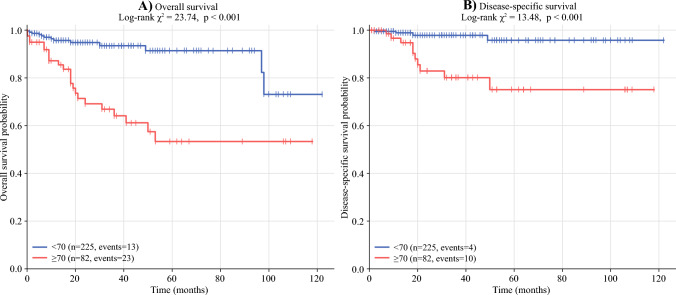
Kaplan–Meier analysis of overall and disease-specific survival by age group. (A) Overall survival and (B) disease-specific survival were compared between patients aged <70 years and ≥70 years. Tick marks indicate censored observations. Survival curves were compared using the log-rank test. Both overall survival and disease-specific survival differed significantly between age groups, with poorer survival observed among patients aged ≥70 years

Over a median follow-up of 17 months (IQR 7–39), 36 deaths occurred among 307 patients with evaluable survival data. Cause of death was explicitly cancer related in four patients, non-cancer related in eight patients, and unknown in 24 patients. Among the 24 patients with unknown cause of death, 10 had documented disease recurrence before death and were therefore included as DSS events according to the prespecified pragmatic DSS definition. Accordingly, 14 deaths were counted as DSS events. Both OS (log-rank *χ*^2^ = 23.7, *p* < 0.001) and DSS (log-rank *χ*^2^ = 13.5, *p* < 0.001) differed between age groups (Fig. 3); median OS was 108.8 months (age < 70 years) versus 73.9 months (age ≥ 70 years), and median DSS was not reached in either group. In stage-stratified DSS analysis, the age difference did not reach significance (stage I–II *p* = 0.088; stage III–IV *p* = 0.072); in histology strata, the age difference was significant for low-grade endometrioid disease (*p* = 0.013) but not high grade (*p* = 0.144). Corresponding OS stratified analyses followed the same pattern with stronger effect sizes, reflecting the contribution of non-cancer mortality. Stratified curves are presented in Supplementary Figs. S1. and S2.

In univariable Cox models for DSS (14 events), age ≥ 70 years (HR 6.57; 95% CI 2.06–20.95; *p* = 0.002), advanced stage (HR 6.05; *p* < 0.001), high-grade histology (HR 6.09; *p* < 0.001), ACCI ≥ 4 (HR 11.12; *p* < 0.001), and ASA III–IV (HR 3.33; *p* = 0.031) were associated with worse DSS; SII was not (HR 1.74, *p* = 0.32). PNI per 5-point increase was protective (HR 0.36; 95% CI 0.19–0.68; *p* = 0.002). In a parsimonious multivariable DSS Cox model (age, stage, PNI), advanced stage remained independently associated with worse DSS (HR 3.26; 95% CI 1.02–10.42; *p* = 0.046), PNI was not significantly associated with DSS (HR 0.51; 95% CI 0.26–1.01; *p* = 0.053), and age ≥ 70 years was no longer independently associated with cancer-related mortality (HR 2.74; *p* = 0.141).

For OS, univariable Cox analyses (36 deaths) identified age ≥ 70 years (HR 4.64; *p* < 0.001), advanced stage (HR 4.50; *p* < 0.001), high-grade histology (HR 5.46; *p* < 0.001), ASA III–IV (HR 9.40; *p* < 0.001), and ACCI ≥4 (HR 7.78; *p* < 0.001) as significant predictors; SII was not. Each 5-point increase in PNI was associated with a 68% reduction in all-cause mortality (HR 0.32; *p* < 0.001). In the multivariable OS Cox model (likelihood ratio *χ*^2^=100.9, *df* = 5, *p* < 0.001; age, stage, histology, ASA, PNI per 5-point increase), higher PNI remained the strongest protective factor (HR 0.48; 95% CI 0.32–0.72; *p* < 0.001) and ASA III–IV was independently associated with worse survival (HR 3.80; *p* = 0.002), whereas chronological age (HR 1.19; *p* = 0.69), advanced stage (HR 2.09; *p* = 0.058), and histologic subtype (HR 1.72; *p* = 0.16) were not. The SII and ACCI were excluded because of the absence of an independent association in univariate analysis and to avoid collinearity with the ASA class, respectively (Table 4).

**Table 4:** Univariate and multivariable Cox proportional hazards regression for overall survival and disease-specific survival

Survival	HR (95% CI)	*p*-Value	HR (95% CI)	*p*-Value
Overall (*n* = 307, 36 events)	Univariate		Multivariable	
Age ≥70 versus <70	4.64 (2.35–9.17)	**< 0.001**	1.19 (0.50–2.86)	0.694
Stage III–IV versus I–II	4.50 (2.34–8.66)	**< 0.001**	2.09 (0.98–4.49)	0.058
High-grade versus low-grade	5.46 (2.79–10.67)	**< 0.001**	1.72 (0.80–3.67)	0.164
ASA III–IV versus I–II	9.40 (4.80–18.40)	**< 0.001**	3.80 (1.61–8.96)	**0.002**
ACCI ≥4 versus 2–3	7.78 (3.73–16.20)	**< 0.001**	–	–
SII high versus low	1.32 (0.68–2.57)	0.409	–	–
PNI (per 5-point increase)	0.32 (0.22–0.45)	**< 0.001**	0.48 (0.32–0.72)	**< 0.001**
Disease-specific (*n* = 307, 14 events)	Univariate		Parsimonious model^a^	
Age ≥ 70 versus < 70	6.57 (2.06–20.95)	**0.002**	2.74 (0.72–10.50)	0.141
Stage III–IV versus I–II	6.05 (2.10–17.45)	**< 0.001**	3.26 (1.02–10.42)	**0.046**
High-grade versus low-grade	6.09 (2.09–17.73)	**< 0.001**	–	–
ASA III–IV versus I–II	3.33 (1.11–9.95)	**0.031**	–	–
ACCI ≥4 versus 2–3	11.12 (3.10–39.95)	**< 0.001**	–	–
SII high versus low	1.74 (0.58–5.21)	0.319	–	–
PNI (per 5-point increase)	0.36 (0.19–0.68)	**0.002**	0.51 (0.26–1.01)	0.053

Disease-specific survival defined as death from endometrial cancer or death from unknown cause with documented recurrence (14 events from 36 total deaths). Bold values indicate statistical significance (*p* < 0.05)

^a^Parsimonious model (three variables: age, stage, PNI) selected due to limited events (*n* = 14, events per variable = 4.7). Full five-variable model showed overfitting with HR reversal for ASA (data not shown)

ACCI, Age-adjusted Charlson comorbidity index; ASA, American Society of Anesthesiologists; CI, confidence interval; HR, hazard ratio; PNI, prognostic nutritional index; SII, systemic immune-inflammation index

## Discussion

In this single-center cohort of 319 women undergoing surgery for endometrial cancer, the principal finding is that chronological age ≥ 70 years was not an independent predictor of either major postoperative morbidity or OS once physiological reserve and nutritional status were accounted for. Instead, low PNI and ASA III–IV class emerged as the most consistent predictors of both major morbidity and survival across multivariable models. These findings support a shift from chronology-based to physiology-based risk stratification in older women with endometrial cancer and align with the broader geriatric oncology paradigm that frailty and nutritional reserve are more informative than age alone.^4,8,12,24^

The strong association of ASA III–IV and low PNI with major morbidity in our multivariable logistic regression (OR 3.89 and 5.78, respectively) is consistent with earlier reports linking ASA class to adverse perioperative outcomes in gynecologic oncology^25,26^ and with the well-established prognostic role of immune-nutritional indices in surgical patients.^6,7,20,22,23^ The biological basis for the PNI effect is plausible: low serum albumin reflects systemic inflammation and catabolism, whereas lymphopenia indicates impaired cell-mediated immunity. Together these mechanisms contribute to impaired wound healing, increased susceptibility to infection, and delayed functional recovery.^27,28^ Notably, in our cohort, postoperative infection was the single most common complication, occurring more than twice as often in older patients (9.7% vs. 4.0%)—mirroring the pattern expected when immunonutritional reserve is lower. In contrast, SII showed only a non-significant numerical trend with major morbidity (*p* = 0.09), in keeping with prior studies in which the prognostic value of the SII has varied according to cohort size and event numbers.^9,29^

Age, advanced stage, and high-grade histology were all significant predictors of mortality in univariable analyses, but their effect attenuated or disappeared after adjustment for stage and PNI. Two caveats deserve emphasis. First, the event count for major morbidity (*n* = 14) and for cancer-related death (*n* = 14) constrains how many covariates can be safely accommodated in multivariable models; we therefore prioritized parsimonious models and report extended sensitivity analyses. Second, comparing OS and DSS clarifies the role of non-cancer mortality. Of 36 deaths with evaluable follow-up, eight were explicitly non-cancer and 14 were classified as cancer-related (four with documented cancer cause and 10 unknown-cause deaths in patients with documented recurrence). The age-related OS difference persisted within stage I–II (*p* < 0.001) and low-grade (*p* < 0.001) strata but attenuated in the corresponding DSS stratified analyses (stage I–II *p* = 0.088; low-grade *p* = 0.013), consistent with non-cancer mortality contributing meaningfully to the OS gap. In the parsimonious multivariable DSS model (age, stage, PNI), age was no longer independently associated with cancer-related mortality (HR 2.74; *p* = 0.14), whereas advanced stage remained significant (HR 3.26; *p* = 0.046) and PNI retained a borderline protective effect (*p* = 0.053). Taken together with the OS results, these findings indicate that physiologic-reserve variables (ASA, PNI) drive overall mortality risk, whereas disease-related variables (stage, PNI) drive cancer-specific mortality, and chronological age does not independently contribute to either after appropriate adjustment.

An important and previously underappreciated finding is the pattern of recurrence in advanced-stage disease. Within stage III–IV, older patients experienced recurrence at nearly three times the rate of younger patients (53.6% vs. 18.8%; *p* = 0.007), whereas no significant difference was observed in stage I–II. In the same stage stratum, adjuvant chemotherapy was administered less frequently to older patients (68.8% vs. 90.6%; *p* = 0.060). Although this does not establish causation, the concurrence of lower chemotherapy exposure with higher recurrence in older patients with advanced disease raises the possibility of undertreatment and warrants further prospective evaluation—particularly of prehabilitation strategies designed to increase chemotherapy tolerance in fit older patients.

Our data also speak directly to the question of whether operative approach is equitably offered across age groups. In early-stage disease, the proportion of minimally invasive procedures did not differ significantly between younger and older patients (39.7% vs. 27.9%; *p* = 0.13), suggesting that minimally invasive surgery is not being disproportionately withheld from older women with operable stage I–II disease in our practice. The substantially lower minimally invasive rate in older patients with stage III–IV disease (37.5% vs. 3.1%; *p* = 0.001) reflects the greater debulking and nodal dissection requirements typical of advanced disease as well as the intersection of comorbidity and high-risk pathology in this subgroup. Our overall major morbidity rate of 3.8% is lower than the 5–29% range reported in earlier perioperative series of older gynecologic oncology patients,^5,30^ which we attribute to patient selection, standardized perioperative protocols, and contemporary anesthetic and supportive care.

Strengths and limitations. Strengths include a well-defined tertiary-center cohort with uniform surgical and pathological protocols, standardized Clavien–Dindo grading, and simultaneous evaluation of nutritional (PNI), inflammatory (SII), and comorbidity (ACCI) indices. The concurrent availability of adjuvant therapy data and stage-stratified analyses allowed the clinically important subgroup findings described above.

Limitations must be acknowledged. The retrospective design carries inherent risks of selection and information bias, and exclusion of patients with incomplete records may introduce bias favoring more complete outcomes. The major morbidity event count (*n* = 14) limits the events-per-variable ratio in logistic regression; results from multivariable modeling of rare outcomes should therefore be confirmed in larger cohorts. Ascertainment of cause-specific mortality in retrospective data is imperfect: of 36 deaths in our cohort, only four were explicitly coded as cancer-related in available records, whereas 24 had an unknown cause; for the primary DSS analysis, we used a pragmatic definition (documented cancer cause or unknown cause with documented recurrence). Finally, the single-center nature of the study and the practice patterns of a high-volume gynecologic oncology unit may limit generalizability to other settings.

Implications and future directions. The data support an integrated preoperative risk-stratification pathway in older women with endometrial cancer that combines ASA class, ACCI, and PNI rather than relying on age alone. The observed recurrence gap in advanced disease and the concurrent gap in chemotherapy exposure highlight the need for prospective studies of prehabilitation, nutritional optimization, and geriatric-assessment-guided adjuvant treatment decision-making. Standardization of PNI and ACCI cutoffs in multicenter cohorts is a natural next step, as is the integration of these indices into treatment planning pathways.

## Conclusion

Older age is associated with more aggressive tumor biology but not with higher rates of major complications or 30-day mortality in surgically treated endometrial cancer. The PNI and ASA class are independent predictors of major morbidity and survival, whereas chronological age is not. These findings support integrating nutritional and physiological assessment rather than age alone into preoperative risk stratification and highlight the need for prospective evaluation of prehabilitation and adjuvant treatment pathways in older women with advanced stage disease.

## Supplementary Information

Below is the link to the electronic supplementary material.

Supplementary file1 (PDF 202 kb)

Supplementary file2 (PDF 223 kb)

## Data Availability

The datasets generated and/or analyzed during the current study are available from the corresponding author on reasonable request.

## Permission to Reproduce Material

Not applicable as no third-party material was reproduced.
