# Supplementary material for: Perioperative Risk Stratification in Surgically Treated Endometrial Cancer: The Impact of Nutritional Status and Comorbidity Burden on Morbidity and Survival
Source: Ann Surg Oncol. 2026 Jun 2;33(9):8358–68. doi: 10.1245/s10434-026-19906-5 (PMC13452849; doi:10.1245/s10434-026-19906-5)

Supplementary Figure S1. Stratified Disease-Specific Survival by Age Group

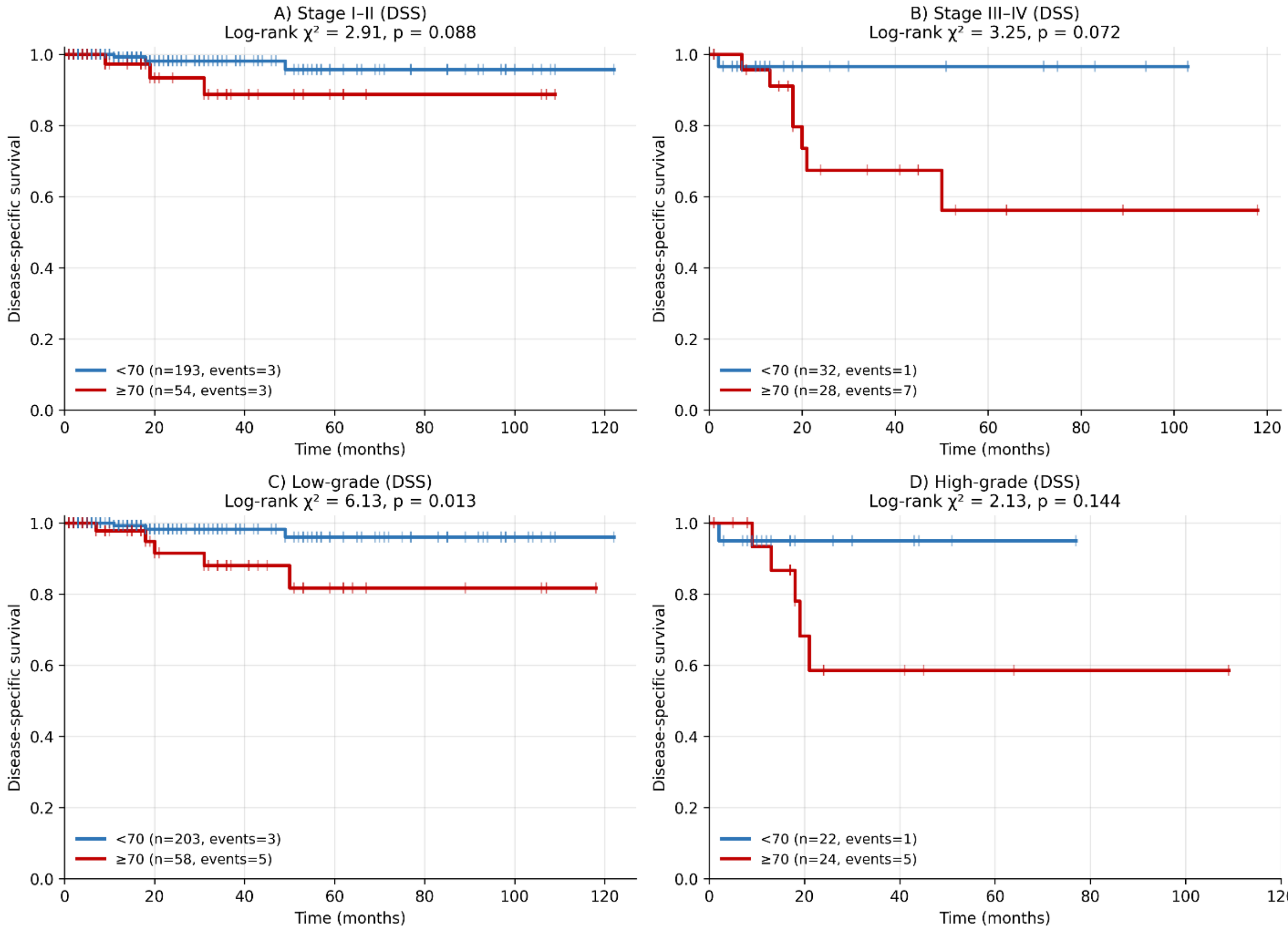

Supplement: Supplementary file 1 [file 10434_2026_19906_MOESM1_ESM.pdf]
